# Genome-wide identification and expression analysis of *GRAS* gene family in *Eucalyptus grandis*

**DOI:** 10.1186/s12870-024-05288-x

**Published:** 2024-06-18

**Authors:** Haifei Lu, Jianmin Xu, Guangyou Li, Tailin Zhong, Danwei Chen, Jiabin Lv

**Affiliations:** 1 College of Urban Construction, Zhejiang Shuren University, Hangzhou, 310015 China; 2 School of Forestry & Landscape Architecture, Anhui Agricultural University, Hefei, 230036 Anhui China; 3 Anhui Province Key Laboratory of Forest Resources and Silviculture, Anhui Agricultural University, Hefei, 230036 China; 4 Key Laboratory of State Forestry Administration on Tropical Forestry, Research Institute of Tropical Forestry, Chinese Academy of Forestry, Guangzhou, 510520 China

**Keywords:** Eucalyptus grandis, GRAS family, Genome-wide analysis, Phytohormone, Abiotic stress

## Abstract

**Background:**

The *GRAS* gene family is a class of plant-specific transcription factors with important roles in many biological processes, such as signal transduction, disease resistance and stress tolerance, plant growth and development. So far, no information available describes the functions of the *GRAS* genes in *Eucalyptus grandis*.

**Results:**

A total of 82 *GRAS* genes were identified with amino acid lengths ranging from 267 to 817 aa, and most *EgrGRAS* genes had one exon. Members of the *GRAS* gene family of *Eucalyptus grandis* are divided into 9 subfamilies with different protein structures, while members of the same subfamily have similar gene structures and conserved motifs. Moreover, these *EgrGRAS* genes expanded primarily due to segmental duplication. In addition, *cis*-acting element analysis showed that this family of genes was involved involved in the signal transduction of various plant hormones, growth and development, and stress response. The qRT-PCR data indicated that 18 *EgrGRAS* genes significantly responded to hormonal and abiotic stresses. Among them, the expression of *EgrGRAS13*, *EgrGRAS68* and *EgrGRAS55* genes was significantly up-regulated during the treatment period, and it was hypothesised that members of the *EgrGRAS* family play an important role in stress tolerance.

**Conclusions:**

In this study, the phylogenetic relationship, conserved domains, *cis*-elements and expression patterns of *GRAS* gene family of *Eucalyptus grandis* were analyzed, which filled the gap in the identification of *GRAS* gene family of *Eucalyptus grandis* and laid the foundation for analyzing the function of *EgrGRAS* gene in hormone and stress response.

**Supplementary Information:**

The online version contains supplementary material available at 10.1186/s12870-024-05288-x.

## Introduction

The *GRAS* gene family encodes a group of plant-specific transcriptional regulators, named after the initial three recognised family members: Gibberellic Acid Insensitive (GAI), Repressor of GA1 (RGA), and Scarecrow (SCR) [1–3]. The majority of GRAS proteins have C-terminal regions that are highly conserved and range in size from 400 to 700 amino acids [4]. The C-terminal sequence often consists of the LHRI, VHIID, LHRII, PFYRE, and SAW motifs [5]. Furthermore, GRAS proteins have changeable sequences at the N-terminal that enable the proteins to adapt their N-terminal structure in order to selectively and flexibly recognize ligands. As a result, the GRAS family performs a variety of functions, such as participating in gibberellins [6], light signals [7], and other signaling pathways [8], regulating the development of meristem [9], root [10], stem and leaf [11], and responding to abiotic stresses that plants experience like salt, drought, and low temperatures.

The SCR, SHR, and DELLA subfamilies of the *GRAS* gene family have been the subjects of most of the related researches since their genes have varied physiological activities. In addition to playing a role in PIF co-activation and serving as moderators of JA signaling, DELLA proteins are important regulators in the GA signaling pathway [12, 13]. Members of the SCR and SHR subfamilies are involved in controlling the radial root growth of *Arabidopsis thaliana* [14]. *AtSCL3* is a tissue-specific integrator of the GA pathway that promotes *Arabidopsis* root cell division and elongation [15]. In addition, the expression of nine *GRAS* genes was up-regulated in *Larix kaempferi* under GA_3_ treatment [16]. Furthermore, *GRAS* genes are involved in the response to a variety of plant adversity stresses. In *Arabidopsis*, over expression of the *Halostachys caspica SCL13* gene accelerated vegetative growth and enhanced chlorophyll content, fresh weight, and root elongation, indicating that *HcSCL13* enhances plant salt tolerance [17]. Similarly, the poplar SCL gene *PeSCL7* was localised in the nucleus, and transgenic *Arabidopsis thaliana* plants showed enhanced tolerance to drought and salt stress [18]. In rice, *OsGRAS23* can enhance antioxidant properties, reduce H_2_O_2_ accumulation, and improve drought tolerance in transgenic rice [19]. Over expression of *VaPAT1* improved cold tolerance, drought tolerance and high salt tolerance in transgenic *Arabidopsis*. DELLA protein not only regulates root hair growth, but also maintains the low level concentration of ROS and improves plant cold tolerance [20]. *GRAS* gene was found to be involved in low temperature stress in banana, pumpkin, tomato and other plants, and its expression increased after low temperature induction [21–23].

*Eucalyptus grandis* is one of the three fastest growing tree species in the world, with the advantages of fast growth rate, short rotation cycle, and good wood quality. It is widely used in pulp and paper, wood processing, medical treatment, spices and other industries, making it of great economic and environmental value [24, 25]. Eucalypts are mainly distributed in subtropical and tropical areas, where temperature limits its distribution. The completion of the whole genome sequencing of *E. grandis* provides favorable conditions for gene cloning, functional analysis, and bioinformatics analysis [26].

*GRAS* is an important plant transcription factor, widely involved in plant growth and development, and plays an important role in abiotic stress response. To enrich the genetic resources of *E. grandis*, 82 *GRAS* genes were identified from the whole genome. Additionally, analyses of their phylogenetic relationships, chromosomal position, promoter, sequence characteristics, *cis*-acting element and collinearity were performed. Furthermore, we investigated the expression patterns of the *EgrGRAS* genes in different tissues. In particular, the expression patterns of 18 representative *EgrGRAS* genes were analyzed by qRT-PCR under low temperature, drought, salt, and hormone treatment. These results can serve as a reference for further elucidating the specific functions of *EgrGRAS* genes in response to abiotic stress and for screening stress resistance-related *EgrGRAS* gene resources.

## Results

### Identification and characterization of the GRAS gene family in *E. grandis*

A total of 91 protein sequences containing GRAS domain were identified in the whole-genome protein database of *E. grandis* using GRAS HMM (PF03514), and *82 GRAS non-redundant proteins were selected manually*. The *EgrGRAS* genes were named *EgrGRAS1* to *EgrGRAS82* based on their physical location on the chromosomes (from top to bottom). Table 1 showed the analysis of the physicochemical characteristics of the *EgrGRAS* genes, including open reading frame (ORF) length, chromosomal location, exons, protein molecular weight (MW) and isoelectric point (pI). The molecular weights of the GRAS proteins ranged from 31.48 kDa to 92.76 kDa, with the highest pI value being 9.15 (EgrGRAS78) and the lowest pI value being 4.86 (EgrGRAS1). They encode proteins with an average size of 577 aa and a size range of 267 to 817 aa. Additionally, subcellular localization analysis revealed that the EgrGRAS proteins was primarily located in the nucleus, and a few proteins located in the chloroplast and cytoplasm. Only EgrGRAS50 located in the mitochondrion (Table S1).

**Table 1:** Details of the identified *GRAS* genes in *E. grandis*

Name	Gene Identifier	Chr	Location	ORF length (bp)	Protein			
					Length (a.a.)	PI	Mol.Wt. (kDa)	Exons
EgrGRAS1	Eucgr.A00354.1	1	6,873,715–6,875,415	1701	566	4.86	63.84	1
EgrGRAS2	Eucgr.A00355.1	1	6,866,274–6,868,071	1578	525	5.21	59.63	1
EgrGRAS3	Eucgr.A00605.1	1	15,958,555–15,960,147	1593	530	4.96	59.78	1
EgrGRAS4	Eucgr.A00751.1	1	13,496,131–13,497,428	1026	341	8.97	38.66	3
EgrGRAS5	Eucgr.A00764.1	1	13,261,573–13,263,270	1020	339	5.68	38.46	1
EgrGRAS6	Eucgr.A00766.1	1	13,242,710–13,244,073	1179	392	8.74	44.33	1
EgrGRAS7	Eucgr.A00769.1	1	12,971,615–12,973,257	1224	407	6.93	45.55	1
EgrGRAS8	Eucgr.A00772.1	1	12,870,215–12,871,713	1254	417	6.31	46.98	1
EgrGRAS9	Eucgr.A00903.1	1	11,149,472–11,151,374	1419	472	5.68	50.25	1
EgrGRAS10	Eucgr.A01279.1	1	22,092,181–22,096,034	2229	742	6.09	82	1
EgrGRAS11	Eucgr.A01051.1	1	30,751,860–30,756,050	1578	525	6.26	58.56	1
EgrGRAS12	Eucgr.A02754.1	1	42,707,250–42,709,726	1956	651	5.98	70.19	1
EgrGRAS13	Eucgr.B02328.1	2	42,197,166–42,200,226	2127	708	5.74	80.13	1
EgrGRAS14	Eucgr.B02329.1	2	42,203,604–42,205,961	2292	763	6.04	86.18	2
EgrGRAS15	Eucgr.B02330.1	2	42,240,952–42,242,931	1935	644	6.62	72.46	2
EgrGRAS16	Eucgr.B02331.1	2	42,275,526–42,278,050	1968	655	6.7	74.88	1
EgrGRAS17	Eucgr.B02335.1	2	42,294,427–42,298,270	2346	781	5.05	88.55	2
EgrGRAS18	Eucgr.B02337.1	2	42,303,690–42,307,827	2370	789	4.97	87.86	1
EgrGRAS19	Eucgr.B02340.1	2	42,327,077–42,328,672	1425	474	8.71	54.23	1
EgrGRAS20	Eucgr.B02342.1	2	42,360,210–42,362,353	1653	550	5.03	62.42	4
EgrGRAS21	Eucgr.B02343.1	2	42,371,028–42,373,162	1980	659	6.04	74.57	2
EgrGRAS22	Eucgr.B02344.1	2	42,381,654–42,384,177	1983	660	5.93	74.86	2
EgrGRAS23	Eucgr.B02345.1	2	42,388,590–42,391,173	26.01	7.67	5	86.48	2
EgrGRAS24	Eucgr.B02346.1	2	42,392,803–42,396,663	2373	790	5.06	88.46	1
EgrGRAS25	Eucgr.B02347.1	2	42,409,990–42,413,054	1587	528	6.5	59.83	7
EgrGRAS26	Eucgr.B02348.1	2	42,416,921–42,419,659	2352	783	5.18	87.42	2
EgrGRAS27	Eucgr.B02349.1	2	42,421,300–42,424,313	2400	799	5.13	89.79	1
EgrGRAS28	Eucgr.B02350.1	2	42,428,454–42,431,895	2313	770	6.33	87.01	1
EgrGRAS29	Eucgr.B03716.1	2	56,838,604–56,840,793	1416	471	5.83	52.68	1
EgrGRAS30	Eucgr.D01435.1	4	18,740,894–18,744,396	1647	548	5.46	61.31	1
EgrGRAS31	Eucgr.D01917.1	4	32,600,897–32,603,418	2019	672	5.75	75.09	1
EgrGRAS32	Eucgr.E01509.1	5	16,602,168–16,605,196	2190	729	6	79.88	1
EgrGRAS33a	Eucgr.E01510.1	5	16,615,299–16,618,470	2145	714	6.19	78.75	2
EgrGRAS33b	Eucgr.E01510.2	5	16,615,298–16,618,470	1992	664	6.25	72.61	2
EgrGRAS34	Eucgr.E03895.1	5	72,526,135–72,529,466	1638	545	5.4	61.33	1
EgrGRAS35	Eucgr.F01003.1	6	14,196,874–14,200,460	1740	579	6.95	65.00	1
EgrGRAS36	Eucgr.F01978.1	6	26,104,114–26,105,565	1452	483	6.46	53.79	1
EgrGRAS37	Eucgr.F02630.1	6	38,398,707–38,400,163	1413	470	7.33	53.98	1
EgrGRAS38a	Eucgr.F03414.1	6	45,653,169–45,656,704	1719	572	5.38	64.35	1
EgrGRAS38b	Eucgr.F03414.2	6	45,653,168–45,656,704	1314	438	5.67	49.55	1
EgrGRAS39	Eucgr.F03523.1	6	46,379,774–46,381,321	1548	515	5.41	57.45	1
EgrGRAS40	Eucgr.F03769.1	6	48,393,002–48,394,414	1416	471	5.08	50.87	1
EgrGRAS41	Eucgr.F04276.1	6	53,528,051–53,531,508	1620	539	4.98	60.60	1
EgrGRAS42	Eucgr.F04460.1	6	57,147,942–57,150,108	1578	525	5.12	58.11	2
EgrGRAS43	Eucgr.G02939.1	7	50,588,034–50,591,238	2241	746	5.7	82.54	1
EgrGRAS44	Eucgr.G02940.1	7	50,603,408–50,606,039	2049	682	5.36	75.89	4
EgrGRAS45	Eucgr.G02941.1	7	50,618,667–50,621,226	2205	734	6.55	81.29	1
EgrGRAS46a	Eucgr.G03258.1	7	53,266,202–53,270,381	1416	471	6.04	53.06	2
EgrGRAS46b	Eucgr.G03258.2	7	53,266,201–53,270,381	1401	467	5.91	52.65	1
EgrGRAS46c	Eucgr.G03258.3	7	53,266,201–53,270,381	1212	404	5.99	45.97	2
EgrGRAS47	Eucgr.H01009.1	8	11,745,518–11,746,885	1299	432	7.65	49.08	2
EgrGRAS48	Eucgr.H01010.1	8	11,750,284–11,751,981	1698	565	5.68	63.63	1
EgrGRAS49	Eucgr.H01257.1	8	18,726,996–18,728,826	1395	464	6.19	51.83	1
EgrGRAS50	Eucgr.H02292.1	8	29,267,565–29,268,884	1320	439	5.28	49.06	1
EgrGRAS51	Eucgr.H03356.1	8	64,037,713–64,040,009	1884	627	5.35	69.08	2
EgrGRAS52	Eucgr.H04039.1	8	54,902,272–54,904,059	1788	595	5.29	66.95	1
EgrGRAS53	Eucgr.H04688.1	8	65,810,551–65,812,077	1491	496	6.58	55.69	2
EgrGRAS54	Eucgr.I00236.1	9	4,855,110–4,856,239	1083	360	5.45	40.84	2
EgrGRAS55	Eucgr.I00704.1	9	14,551,884–14,553,855	1485	494	5.57	55.80	1
EgrGRAS56	Eucgr.I01625.1	9	26,163,164–26,165,367	2079	692	5.5	76.14	2
EgrGRAS57	Eucgr.I02056.1	9	30,338,844–30,340,098	1053	350	5.91	39.03	1
EgrGRAS58	Eucgr.I02451.1	9	34,995,730–34,998,186	1785	594	5.72	63.93	2
EgrGRAS59	Eucgr.J01242.1	10	14,097,193–14,099,907	2163	720	4.97	80.55	1
EgrGRAS60	Eucgr.J01244.1	10	14,050,253–14,053,115	2121	706	5.21	78.86	1
EgrGRAS61	Eucgr.J02040.1	10	25,658,773–25,660,800	2031	676	6.34	75.70	2
EgrGRAS62	Eucgr.J02041.1	10	25,661,643–25,665,560	2454	817	8.49	92.76	1
EgrGRAS63	Eucgr.J02042.1	10	25,671,693–25,674,698	2298	765	6.01	86.28	1
EgrGRAS64	Eucgr.J02043.1	10	25,701,545–25,703,953	2142	713	5.35	80.22	2
EgrGRAS65	Eucgr.J02044.1	10	25,720,925–25,724,209	2292	763	6.26	86.09	1
EgrGRAS66	Eucgr.J02815.1	10	33,582,088–33,584,072	1677	558	6	61.14	2
EgrGRAS67	Eucgr.K00873.1	11	10,991,091–10,992,542	1455	484	5.63	54.34	1
EgrGRAS68	Eucgr.K01320.1	11	16,575,167–16,578,424	2346	781	5.85	83.65	4
EgrGRAS69	Eucgr.K01383.1	11	17,239,463–17,240,607	882	293	7.66	33.04	2
EgrGRAS70	Eucgr.K01384.1	11	17,252,135–17,252,938	804	267	9.06	31.48	1
EgrGRAS71	Eucgr.K02752.1	11	35,036,106–35,037,545	1356	451	7.14	49.78	2
EgrGRAS72	Eucgr.K02964.1	11	37,078,231–37,079,688	1281	426	5.03	47.73	3
EgrGRAS73	Eucgr.K02965.1	11	37,083,390–37,084,951	1527	508	5.2	57.22	2
EgrGRAS74	Eucgr.K03117.1	11	39,073,889–39,075,865	1977	658	6.52	75.72	1
EgrGRAS75	Eucgr.K03118.1	11	39,093,399–39,095,264	1866	621	9.02	71.15	1
EgrGRAS76	Eucgr.K03119.1	11	39,134,339–39,140,174	1968	655	8.49	75.47	2
EgrGRAS77	Eucgr.K03122.1	11	39,154,812–39,156,638	1830	609	8.99	69.82	1
EgrGRAS78	Eucgr.K03126.1	11	39,219,627–39,221,716	1863	620	9.15	70.96	1
EgrGRAS79	Eucgr.K03127.1	11	39,227,025–39,229,156	1884	627	7.94	71.95	1
EgrGRAS80	Eucgr.K03128.1	11	39,243,476–39,245,578	2073	690	7.02	78.62	2
EgrGRAS81	Eucgr.L02817.1	scaffold_1442	4995–6980	1779	592	6.85	68.37	2
EgrGRAS82	Eucgr.L03490.1	scaffold_3358	978–2707	1491	496	5.7	54.72	2

### Phylogenetic analysis of GRAS gene family

**Fig. 1:**
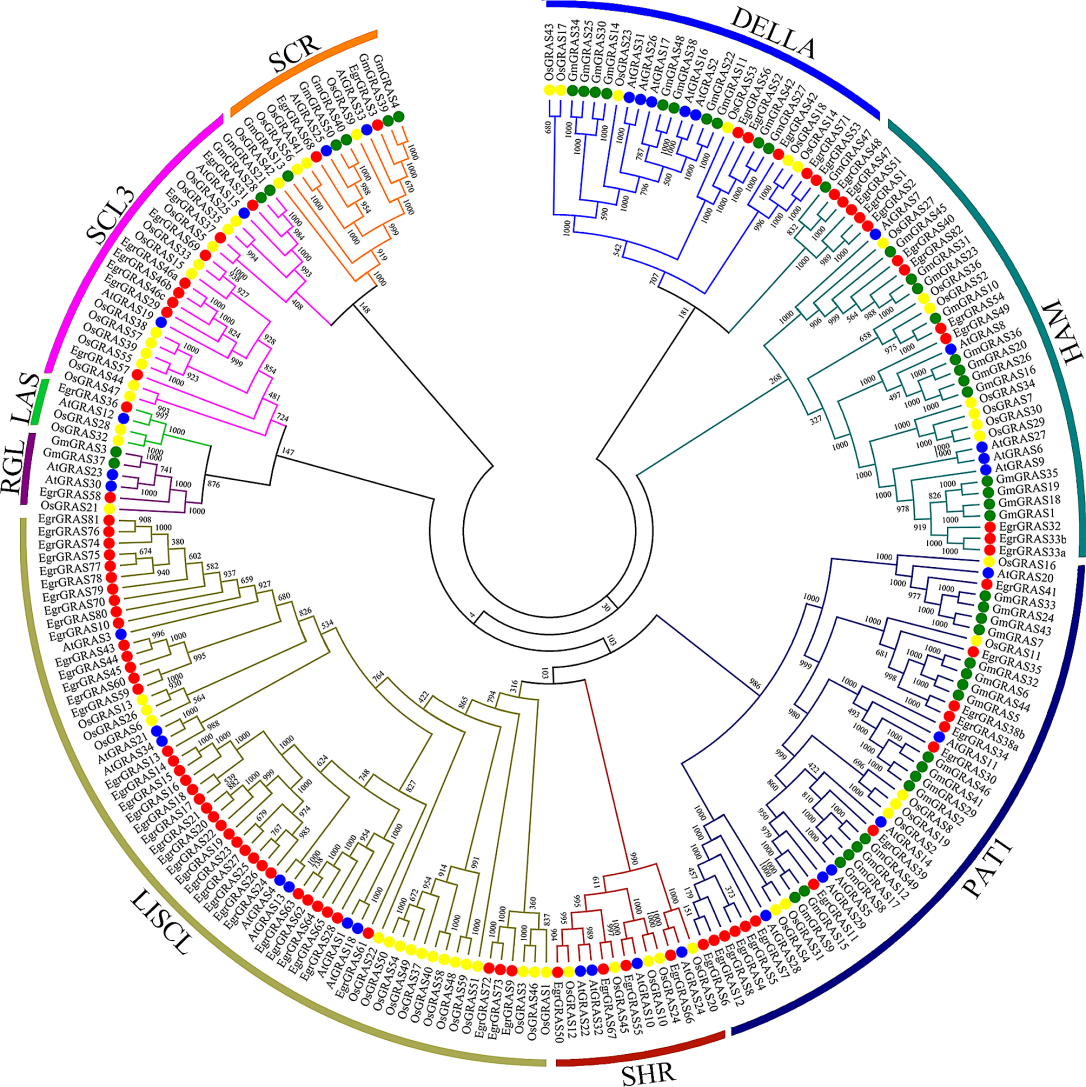
Phylogenetic tree of GRAS genes from E. grandis, Arabidopsis, rice and soybean. 83 *EgrGRAS* genes, 34 *AtGRAS* genes, 40 *OsGRAS* genes and 61 *GmGRAS* genes are clustered into 9 subfamilies. *GRAS* genes from *E. grandis*, *Arabidopsis*, rice and soybean are denote by red, blue, yellow and green shape, respectively. Details of the *GRAS* genes from four species are listed in Table S3. The tree was generated with the Clustal X 2.0 software using the neighbor-joining (N-J) method

To study the relationship and classification of *GRAS* family members in *E. grandis*, an evolutionary tree was constructed with 225 protein sequences including 34 AtGRASs, 59 OsGRASs, 50 GmGRASs and 82 EgrGRASs (Fig. 1 and Table S3). Based on the subfamily classification of the *GRAS* gene family in *Arabidopsis* [27], rice [28] and soybean [29], *GRAS* genes were divided into nine subfamilies: PAT1, SHR, LISCL, HAM, SCR, RGL, LAS, DELLA and SCL3. The 82 *GRAS* genes in *E. grandis* were unevenly distributed among subfamilies. The LISCL subfamily was the largest subfamily with 36 *EgrGRAS* members, followed by the HAM and PAT1 subfamilies with 14 and 13 members respectively, and the LAS and RGL subfamilies were the smallest with only one member. In addition, *EgrGRAS42*, *EgrGRAS53* and *EgrGRAS71* are not classified.

### Gene structure, conserved motif, and multiple alignment analysis

**Fig. 2:**
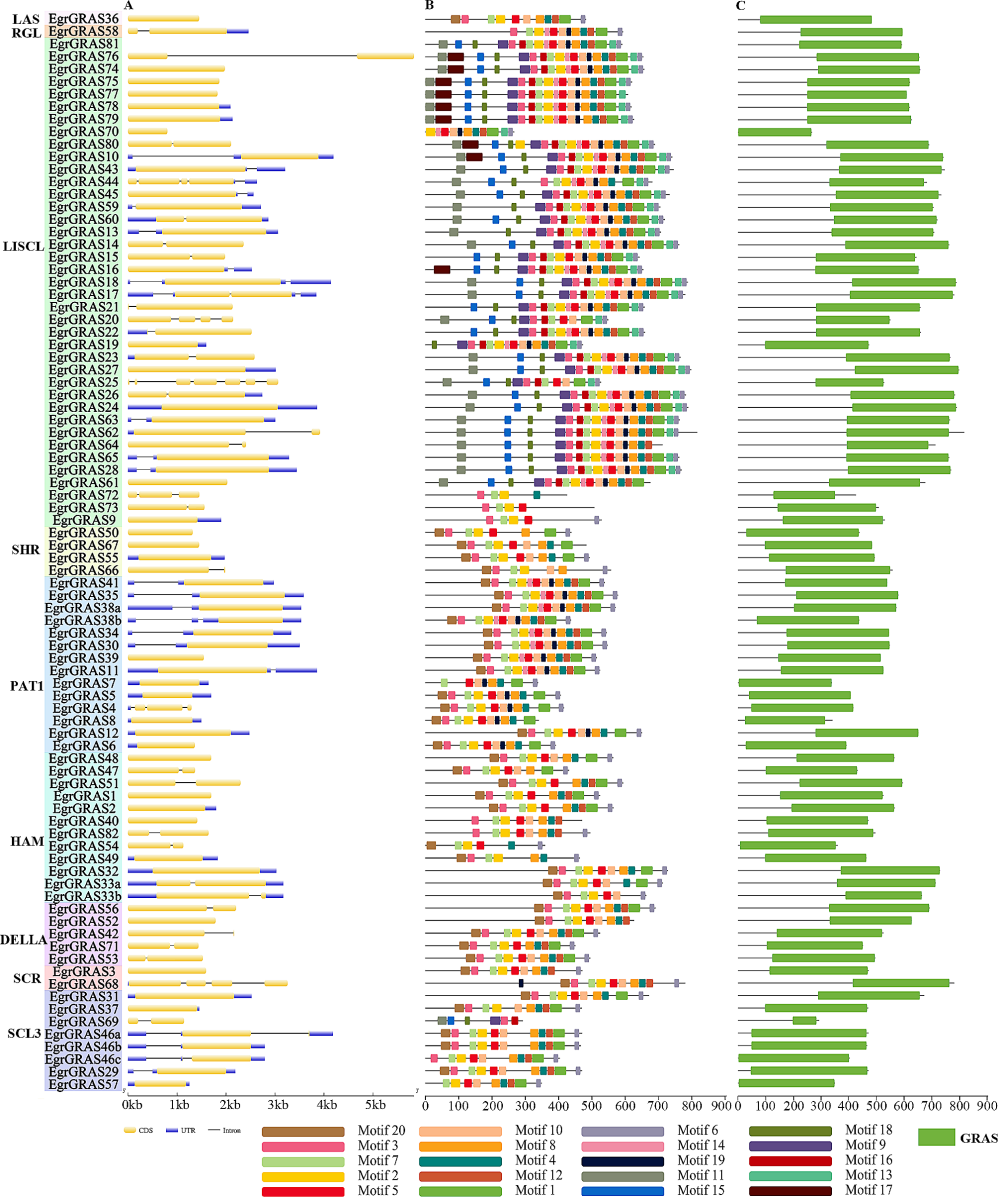
Analysis of the motif and gene structure of the GRAS gene family in *E. grandis*. (**A**) Gene structure of *GRAS* genes in *E. grandis*. Exons are indicated by green rectangles. Gray lines connecting two exons represent introns. (**B**) Conserved motifs of *GRAS* genes in *E. grandis*. Distribution of the 20 conserved motifs in the *EgrGRAS* genes following analysis by MEME tool. The different-colored boxes represent different motifs and their position in each protein sequence of GRAS. (**C**) Domain analysis of GRAS proteins in *E. grandis*

To understand the gene structure of the *GRAS* genes of *E. grandis*, intron-exon structure analysis was performed (Fig. 2A). The results showed that 62.2% (51) of *GRAS* genes had no introns, and *EgrGRAS62* and *EgrGRAS76* had longer introns in the *EgrGRAS* genes with introns. The number of exons in most of *EgrGRAS* genes ranged from 1 to 4. According to Fig. 2A, most of *EgrGRAS* genes possess only one or two exons. Several *EgrGRAS* genes possess multiple exons, like *EgrGRAS25*.

Moreover, MEME tool was used to analyze 82 *GRAS* genes family members of *E. grandis*, and TBtools was used to visualize the conserved motif of *EgrGRAS* genes (Fig. 2B). A total of 20 conserved motifs were identified to identify common motifs between different GRAS proteins (Table S4). The results showed that the number of conserved motifs on each protein ranged from 4 to 20, and most of the conserved motifs existed in the C-terminal domain. For example, Motif 4, Motif 13, Motif 12, Motif 1, Motif 6, and Motif 8. Moreover, Motif 14, Motif 13, Motif 18, and Motif 19 existed primarily in the LISCL subfamily. It was further found that EgrGRAS proteins of the same subfamily had similar motif composition. For example, members of the DELLA subfamily contain only Motif 12, Motif 3, Motif 7, Motif 2, Motif 5, Motif 8, Motif 20, Motif 9, Motif 4, Motif 15, Motif 1, Motif 11, and Motif 6. Compared to the DELLA subfamily, the PAT1 subfamily has more Motif 19. Members of the PAT1 subfamily shared similar motif composition and distribution. In addition, the position and order of the motifs were similar within the same subfamily, but the arrangement of motifs was different among different subfamilies.

As expected domain analysis showed that GRAS domains were mainly distributed in the C-terminal region (Fig. 2C). Further analysed by sequence alignment (Figure S1), the conserved domains could be classified into five domains: LHRI (Motif 12 and Motif 3), VHIID (Motif 7, Motif 2, and Motif 10), LHRII (Motif 5 and Motif 8), PFYRE (Motif 9, Motif 4, and Motif 15). SAW (Motif 1, Motif 11, Motif 20 and Motif 6). However, not all domains are conserved in all members. For example, *EgrGRAS20* and *EgrGRAS25* lack the PFYRE domain, while *EgrGRAS57* and *EgrGRAS70* lack the LHRI domain.

### Chromosomal locations, duplication events, collinearity analysis of EgrGRAS genes analysis

**Fig. 3:**
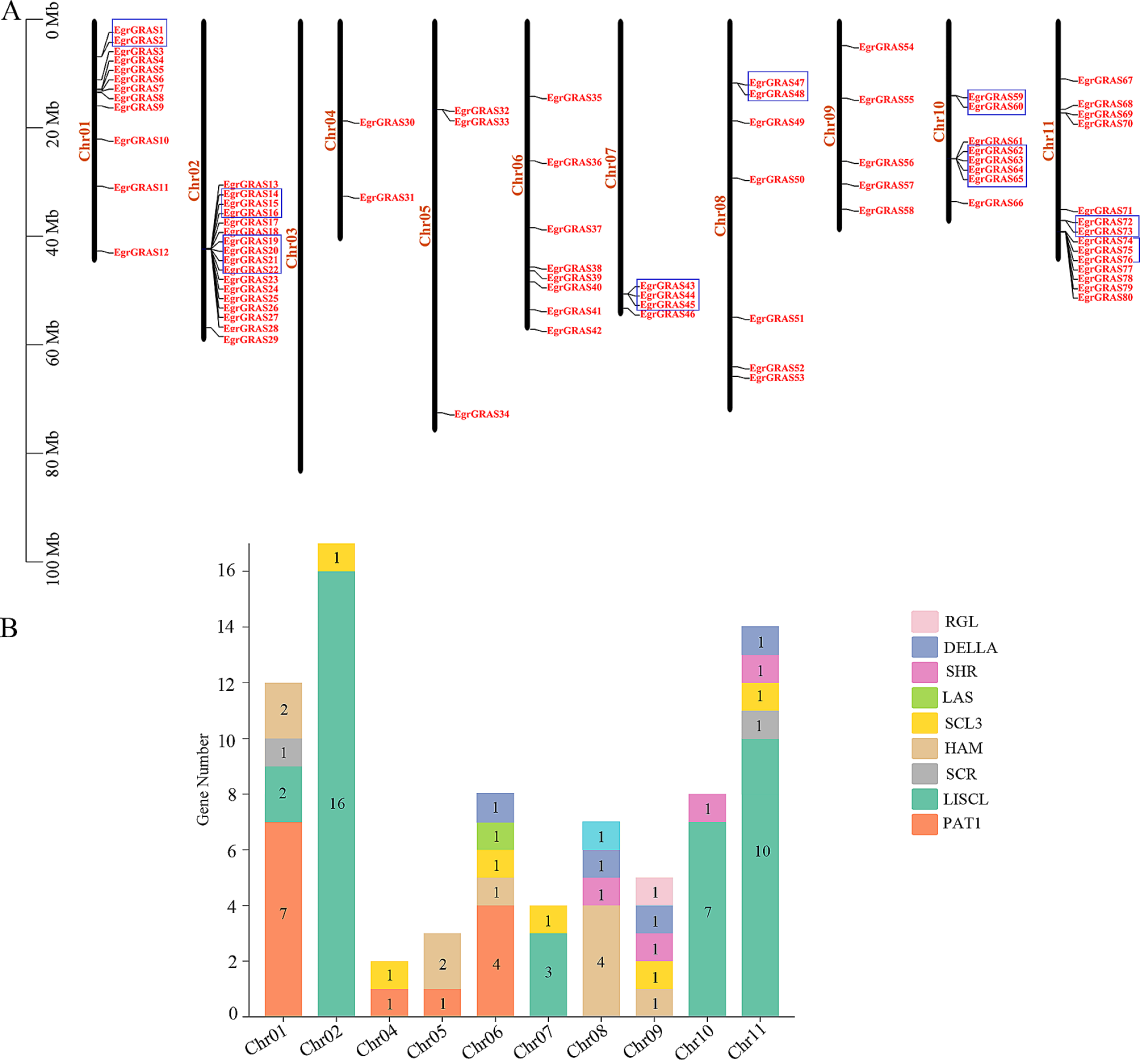
Chromosomal location of *GRAS* genes in *E. grandis*. (**A**) The 82 *GRAS* genes are widely mapped to 11 chromosomes of *E. grandis*. The blue boxes in front of the genes on behalf of these genes belonging to a gene cluster. (**B**) The number distribution of *GRAS* gene family in 10 chromosomes

The chromosomal localization of *GRAS* genes was mapped based on the physical location of the genes in the *E. grandis* genome (Fig. 3A). The results showed that 80 *GRAS* genes were unevenly distributed on 10 chromosomes, and two *GRAS* genes (*EgrGRAS81* and *EgrGRAS82*) were not located on chromosomes, on scaffold_1442 and scaffold_3358 respectively. Most of these *EgrGRAS* genes are distributed on Chr01, Chr02 and Chr11, with 12, 17 and 14, respectively, while the number of genes on Chr04, Chr05 and Chr09 ranges from 2 to 5. As shown in Fig. 3B, Chr11 contains 6 subfamilies of the *EgrGRAS* gene family, Chr06 and Chr09 contain 5 subfamilies of the *EgrGRAS* gene family, whereas Chr02, Chr04, Chr05, Chr07 and Chr10 contain only 2 subfamilies each.

Analysis of chromosomal localization revealed the presence of tandem duplications on Chr01, Chr02, Chr07, Chr08, Chr10 and Chr11, and a total of 24 tandem repeat genes were found (Table S5). All Ka/Ks ratios for duplicated gene pairs were smaller than 0.95, indicating that these genes were subjected to purifying selection. Furthermore, a total of 54 paralogues were identified in *E. grandis GRAS* genes family. All paralogues exhibited a Ka/Ks ratio of less than 1, with the majority falling between 0.1 and 0.5. Further details can be found in the attached Table S5.

**Fig. 4:**
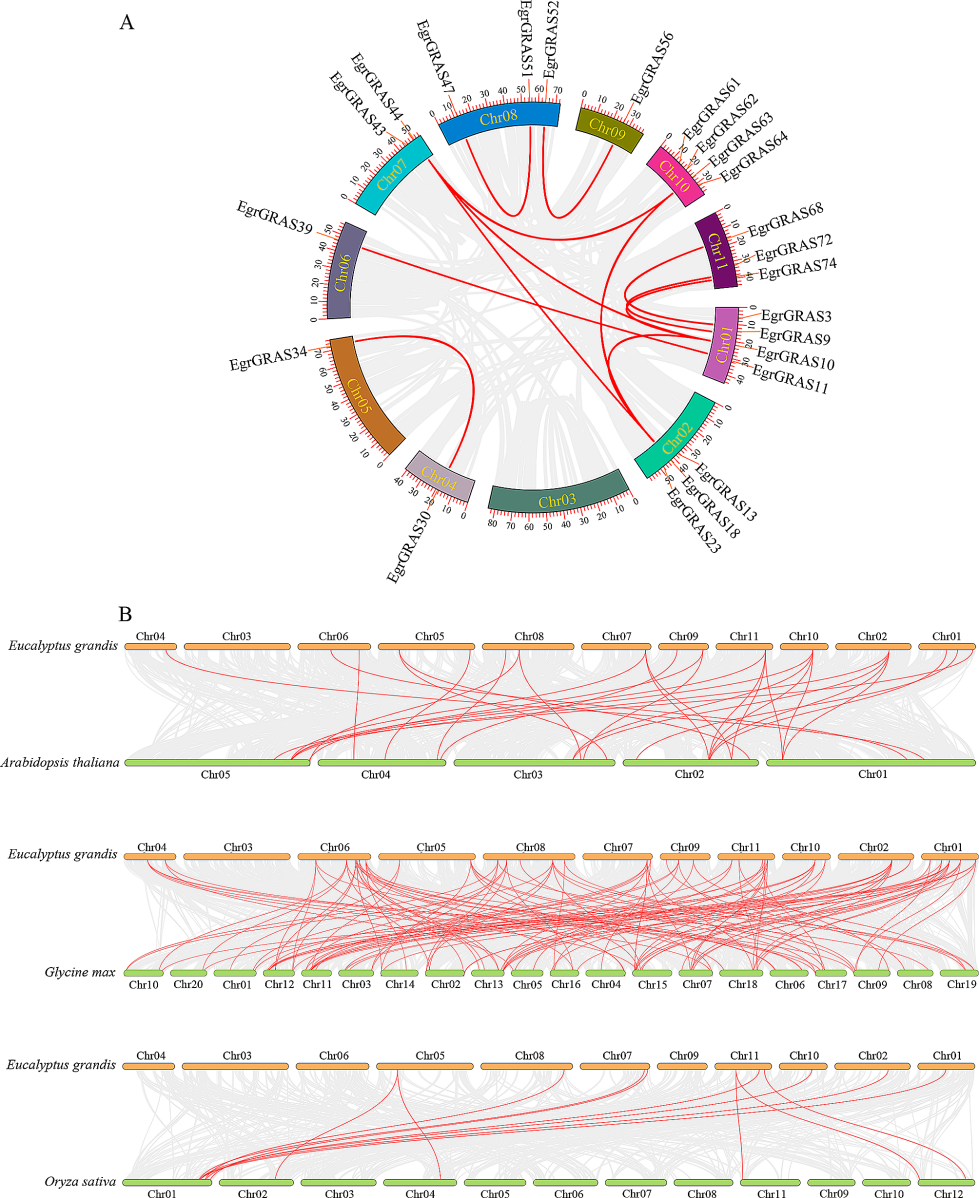
Collinearity analysis. (**A**) Collinearity analysis of *GRAS* gene in *E. grandis*. (**B**) *GRAS* gene collinearity between *E. grandis* and other species (*Arabidopsis*, rice and soybean) genomes

Collinearity analysis was carried out for four plants in order to determine the orthologous relationships of *GRAS* genes between various species. A total of 14 collinearity pairs of 82 *EgrGRAS* genes were obtained with the MCScanX method and no tandem repeat genes (Fig. 4A). Among them, eight pairs of homologous genes in the LISCL subfamily, two pairs in the PAT1 and HAM subfamilies, one pair in the SCR and DELLA subfamilies. Fig. 4B showed that there were many collinear blocks between the genomes of *Arabidopsis*, rice, soybean, and *E. grandis*. Among these blocks, a total number 16, 10, and 42 *EgrGRAS* genes showed pairwise synteny with genes in the *Arabidopsis*, rice, and soybean genome, respectively. This showed that there were more homologous pairs between GRAS genes in *Eucalyptus grandis* and *Arabidopsis thaliana* than those in *Eucalyptus grandis* and rice, as well as a closer evolutionary relationship with soybean. Furthermore, the 6 *EgrGRAS* genes (*EgrGRAS11*, *EgrGRAS13*, *EgrGRAS32*, *EgrGRAS43*, *EgrGRAS61*, and *EgrGRAS74*) were identified to have orthologous genes within other three species genome, simultaneously.

### *Cis*-elements analysis in *EgrGRAS* promoter regions

**Fig. 5:**
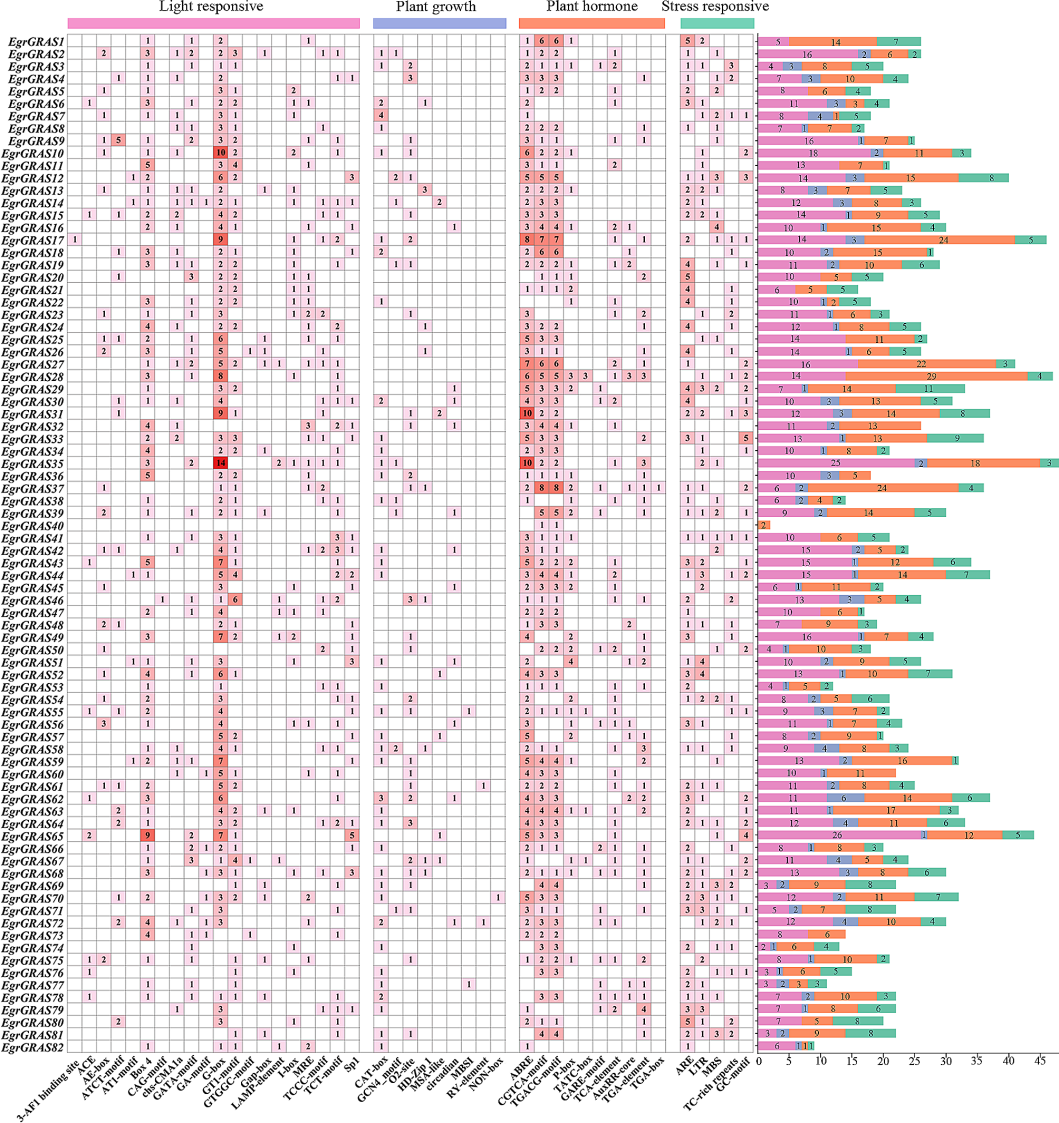
*Cis*-acting elements analysis of *EgrGRAS* genes in promoter region of *E. grandis*. Left panel: Number of each *cis*-acting element in the promoter region (2000 bp) of *EgrGRAS* genes. Right panel: Statistics for the total number of *EgrGRAS* genes

The upstream 2 000 bp promoter region of 82 *EgrGRAS* gene was analyzed, and 2071 elements were obtained, including light, growth development, hormone, and stress response elements. As shown in the Fig. 5, light response elements were found in all promoter regions of the *EgrGRAS* gene except *EgrGRAS40*. And the number of light response elements was the largest, accounting for 40% of all elements. The hormone-related *cis*-acting regulatory elements included abscisic acid-responsive element, MeJA-responsive element, gibberellin-responsive element, salicylic acid-responsive element, and auxin-responsive element. The results revealed that 88% of *EgrGRAS* promoter regions possessed the ABRE elements, suggesting that most of *E. grandis GRAS* genes were promising to be involved in ABA signal pathway. The MeJA-responsive element is also a common *cis*-acting element in promoters, with 83% of *EgrGRAS* genes having both CGTCA-motif and TGACG-motif. In addition, gibberellin-responsive element, auxin-responsive element, and salicylic acid-responsive element were found in 39, 43 and 39 *EgrGRAS* genes promoters, respectively. Five *cis*-elements were related to stress responses including ARE, LTR, MBS, TC-rich repeats, and GC-motif. Notably, more than half of the *EgrGRAS* genes had the low-temperature responsive element. In addition, 136 elements related to plant growth and development were found in promoter regions of *EgrGRAS* genes, among which *cis*-acting regulatory element related to meristem expression (CAT-box) and zein metabolism regulation (O_2_-site) accounted for 66%. MBSI was a *cis*-element of flavonoid biosynthesis gene regulation found only in *EgrGRAS55* and *EgrGRAS77*. This showed that the *EgrGRAS* genes may play an important role in growth and development process and stress response.

### Expression analysis of the *EgrGRAS* in response to hormone and abiotic stresses

According to the results of bioinformatics analysis, 1–3 *EgrGRAS* genes from each subfamily were selected and their expression patterns were analysed after 1, 6, 12, 24 and 168 h treatment with GA_3_, ABA, 4, NaCl and PEG-6000, respectively. The results showed that all 18 *EgrGRAS* genes were responsive to GA_3_, ABA and abiotic stresses, but their expression levels were different.

**Fig. 6:**
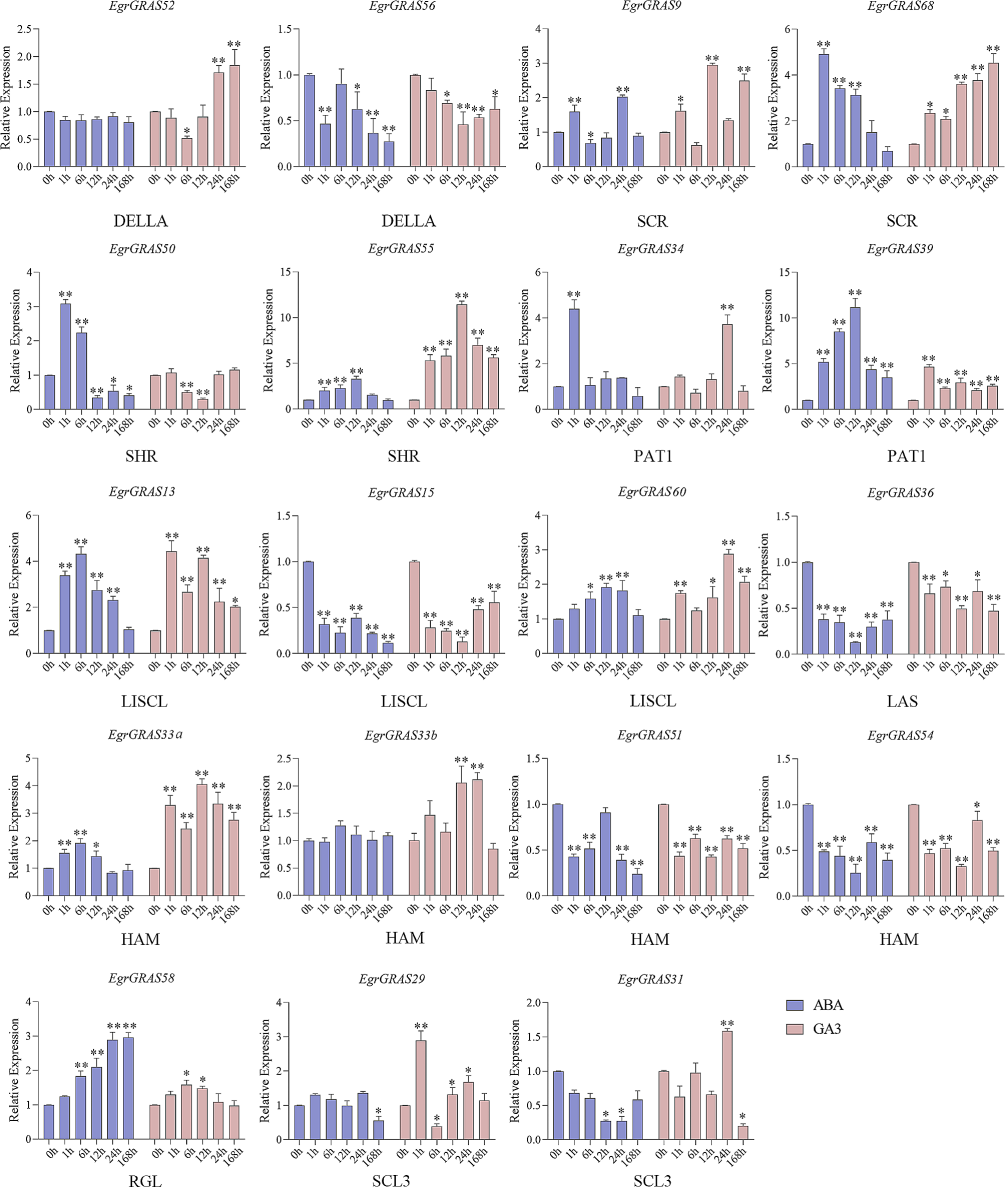
Expression analysis of 18 *EgrGRAS* genes following ABA and GA_3_ treatments by qRT-PCR. The Y-axis and X-axis indicates relative expression levels and the time courses of stress treatments, respectively. Statistical significance was performed using a paired Student’s *t* test. Mean values and standard deviations (SDs) were obtained from three biological and three technical replicates, and significant differences relative to controls were indicated at ^∗^*P* ≤ 0.05 and ^∗∗^*P* ≤ 0.01. The error bars indicate standard deviation

Firstly, to research the response to phytohormone, we determined the expression patterns of *E. grandis GRAS* genes under ABA and GA_3_ treatment. As shown in Fig. 6, more than half (11/18) the *EgrGRAS* genes were up-regulated during ABA treatment, with only *EgrGRAS51* showing decreases after ABA treatment. Among them, the expression of *EgrGRAS68*, *EgrGRAS34* and *EgrGRAS13* were significantly up-regulated and more than 4-fold higher than those of the control group during a treatment period. However, seven *EgrGRAS* genes (*EgrGRAS56, EgrGRAS15*, *EgrGRAS36, EgrGRAS51, EgrGRAS54*, *EgrGRAS29*, and *EgrGRAS36*,) were apparently down-regulated under ABA treatment all the time. Furthermore, all the analyzed genes exhibited differential expression in response to GA_3_ treatment. The expression levels of *EgrGRAS68*, *EgrGRAS55*, *EgrGRAS39*, *EgrGRAS13* and *EgrGRAS33* were significantly and continuously up-regulated. Significantly, expression of *EgrGRAS55* peaked at 12 h under ABA and GA_3_ treatment and were strongly up-regulated (more than 10-fold) in response to GA_3_ treatment. Specifically, the expression levels of *EgrGRAS56*, *EgrGRAS15*, *EgrGRAS36*, *EgrGRAS52* and *EgrGRAS54* were continuously inhibited under ABA and GA_3_ treatment. In addition, it was found that the expression pattern of *EgrGRAS68* under the two hormone treatments was different. It reached the maximum value at 1 h after ABA treatment and then decreased, while it showed a gradual increasing trend after GA_3_ treatment.

**Fig. 7:**
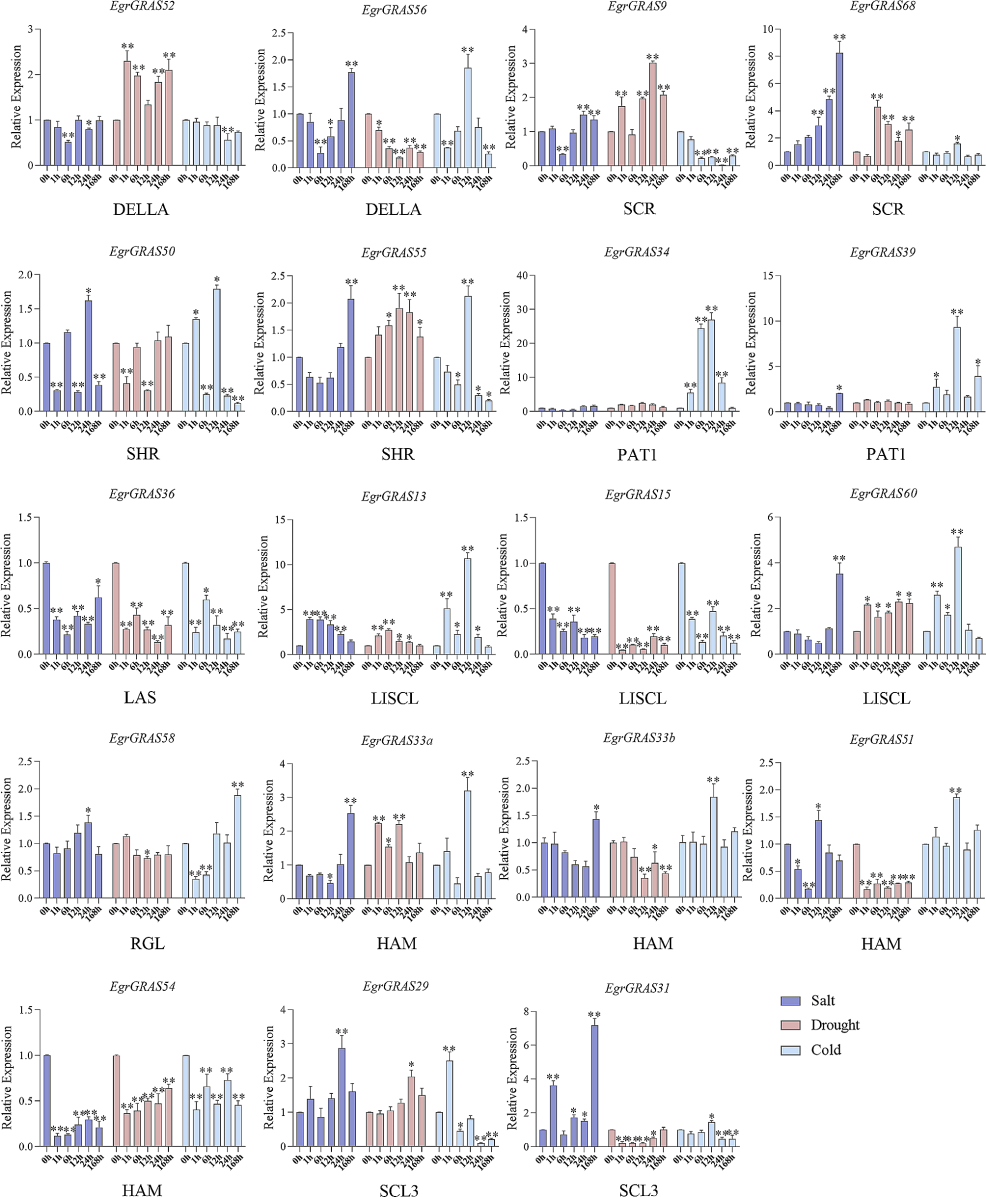
Expression analysis of 18 *EgrGRAS* genes following cold, salt and drought treatments by qRT-PCR. The Y-axis and X-axis indicates relative expression levels and the time courses of stress treatments, respectively. Statistical significance was performed using a paired Student’s *t* test. Mean values and standard deviations (SDs) were obtained from three biological and three technical replicates, and significant differences relative to controls were indicated at ^*^P ≤ 0.05 and ^**^P ≤ 0.01. The error bars indicate standard deviation

Furthermore, we analyzed the expressions of *EgrGRAS* genes in response to NaCl, PEG and low-temperature treatments. Under 300 mM NaCl treatment, the expression of 18 *GRAS* genes in *E. grandis* were shown in Fig. 7. The expression levels of *EgrGRAS9*, *EgrGRAS50*, *EgrGRAS58* and *EgrGRAS29* peaked at 24 h after salt stress, while 7 *EgrGRAS* genes (*EgrGRAS56, EgrGRAS68, EgrGRAS55, EgrGRAS60, EgrGRAS33a, EgrGRAS33b, and EgrGRAS31*) were upregulated to the maximum at 168 h. As for the drought stress, most of the *EgrGRAS* genes were significantly induced at different time points under PEG treatment, but *EgrGRAS39* and *EgrGRAS58* were stably expressed. Among them, *EgrGRAS33*, *EgrGRAS51*, *EgrGRAS59* and *EgrGRAS53* showed a rapid response, reaching the peak after 1 h of drought stress. The expression levels of *EgrGRAS68, EgrGRAS55, EgrGRAS34* and *EgrGRAS13* were up-regulated, reaching a peak at 6–12 h, and then down-regulated at a later stage. Next, we analyzed the expression of *EgrGRAS* genes under cold stress. The expression pattern of 11 genes was found to be up-regulated to a peak and then down-regulated. The expression levels of *EgrGRAS55* and *EgrGRAS56* were strongly up-regulated, peaking at 12 h after low-temperature treatment and then significantly down-regulated. Additionally, three genes (*EgrGRAS13, EgrGRAS34*, and *EgrGRAS39*) were strongly up-regulated (more than 10-fold) in response to low-temperature treatment. Overall, all analysed genes showed differential expression in response to at least two abiotic stress treatments. Only *EgrGRAS13* showed increased expression in response to all three stresses, but *EgrGRAS36, EgrGRAS15* and *EgrGRAS54* showed significantly decreased expression in response to any of the three stresses.

### Correlations and coregulatory networks of *EgrGRAS* genes

**Fig. 8:**
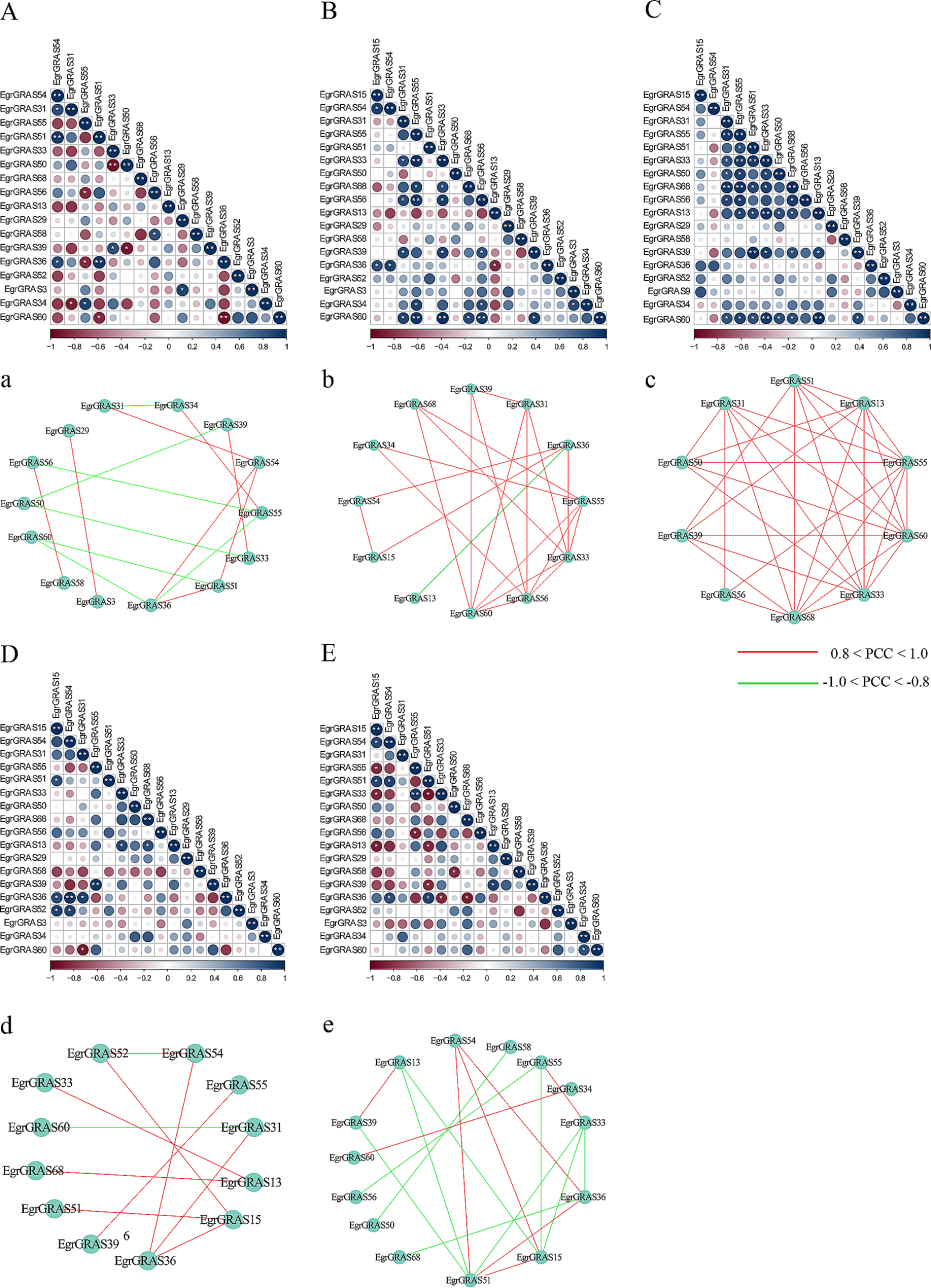
Correlations and co-regulatory networks of 18 *EgrGRAS* genes under stress treatments. (**A**, **B**, **C**, **D**, **E**) Correlation analysis using the R package program. Each correlation is shown by the shades of blue and red and the size of the circle shape. * and ** represent correlations with Pvalue≤0.05 and Pvalue≤0.01, respectively. (**a**, **b**, **c**, **d**, **e**) Co-regulatory networks. The co-regulatory networks of 18 EgrGRAS genes under stress treatments were established based on the Pearson correlation coefficients (PCCs) of these gene pairs using transformed qPCR data

Based on the calculation of PCC values for the relative expression levels of these genes to predict interactions, correlation and coregulatory networks were established. As shown in the Fig. 8, these genes were positively or negatively correlated with each other to varying degrees under different stress treatments. Under PEG treatment, eight gene pairs showed positive correlations (p-value ≤ 0.05 and 0.8 < PCC < 1.0), whereas seven gene pairs showed negative correlations. Among them, *EgrGRAS36* and *EgrGRAS54, EgrGRAS34* and *EgrGRAS55*, and *EgrGRAS39* and *EgrGRAS33* also showed positive correlations under the salt stress. Moreover, all gene pairs showed positive correlations in the cold-related coregulatory networks (p-value ≤ 0.05 and 0.8 < PCC < 1.0). In the co-regulatory network, *EgrGRAS55*, *EgrGRAS59* and *EgrGRAS33* are the hub genes with the highest number of edges. In addition, 18 gene pairs and 10 gene pairs showed significant correlations (p-value ≤ 0.05, -1.0 < PCC < -0.8, 0.8 < PCC < 1.0) under GA and ABA treatments, respectively. It could be found that the *EgrGRAS52*-*EgrGRAS15* and *EgrGRAS36*-*EgrGRAS54* pairs exhibited significant positive correlations under both ABA and GA treatments.

## Discussion

*GRAS* transcription factors are now widely found in plants and can not only participate in light signal transduction and phytohormone signal transduction during plant growth and development, but also play important roles in biotic and abiotic stresses [30]. At present, based on the development of whole genome sequencing technology, the *GRAS* gene family has been identified in many plants, such as *Avena sativa*, *Larix kaempferi* and radish [16, 31, 32].

In this study, a total of 82 *EgrGRAS* family members were identified from the *E. grandis* genome, and the number of genes was much higher than that of European pear (59), peach (48) and *Larix kaempferi* (11) [16, 33]. Similar to previous studies [34], the number of family members is not related to genome size, but may be related to gene duplication events. The 82 *EgrGRAS* genes were classified into nine subfamilies based on evolutionary relationships, including PAT1, SHR, LISCL, HAM, SCR, RGL, LAS, DELLA and SCL3. Consistent with the previous reports, the LISCL and SHR subfamilies did not contain *GRAS* genes from soybean [35]. The phylogenetic tree showed that most of the *EgrGRAS* proteins were classified into the same evolutionary branches as *Arabidopsis* or soybean, suggesting homology in their evolutionary relationships.

Lu et al. found that 54.05% of the cucumber *GRAS* genes family had no introns, and the rest of the genes had only one or two introns [36]. In this study, 82 *EgrGRAS* genes had varying numbers of introns, and 62.2% of the *EgrGRASS* genes lacked introns. The *EgrGRAS* genes belonging to the same subfamily shared similar gene structures, but *EgrGRAS9* and *EgrGRAS68* had large differences in gene structure, which were presumably caused by the loss or addition of introns during the evolutionary process of the genes. The *GRAS* gene family had a relatively conservative evolutionary trend in different species [29, 37, 38], and genes belonging to the same subfamily may have similar functions. It can be seen in Fig. 2 that members of the same subfamily had similar conserved motifs, indicating that members of the same subfamily had similar functions. Except *EgrGRAS5*, *EgrGRAS557*, *EgrGRAS69* and *EgrGRAS570*, almost all members contained motifs 3, 7 and 2, indicating that these motifs played an important role in the *GRAS* gene family, and motifs 17 and 19 were unique to the LISCL subfamily. Differences in motif distribution among subfamilies suggested that these genes may have diverged in function during evolution.

Gene expansion is one of the most important drivers of genome evolution and one of the main reasons for the generation of genes with new functions [39, 40]. And whole-genome duplication and tandem duplication are two important gene expansion pathways, which are prevalent in the process of biological evolution [41]. It had been demonstrated that in rice, apple, and *Arabidopsis thaliana*, gene duplication events promoted the expansion of the *GRAS* genes family [27]. Based on chromosome mapping, we found 24 tandem repeat genes in *E. grandis.* And 14 duplication events were identified, the most duplication events were observed in the LISCL subfamily, while there were two duplication events in the HAM subfamily and one in each of the SCR, PAT1 and DELLA subfamilies. It was found that the most duplication events were observed in the SCL subfamily of *Avena sativa* [31], while the most duplication events were present in the SHR subfamily of *Medicago truncatula* [42]. Tandem duplicated gene pair have similar structure and motif pattern. For example, *EgrGRAS59* and *EgrGRAS60* shared similar features in the exon/intron structure and conserved motifs. These results show that duplication events have contributed to the expansion of the *GRAS* genes family in *E. grandis*, and that duplication events of different subfamilies play distinct important roles in other plants.

In this study, the promoter region of *EgrGRAS* genes has multiple *cis*-acting elements related to hormone or stress response. The expression levels of 18 *EgrGRAS* genes were significantly different after various treatments with gibberellin, abscisic acid, salt, drought, and temperature stresses, and the expression patterns of the same subfamily members were also significantly different. Similar results were found for *GRAS* genes in *Brachypodium distachyon*, tea, and *Phoebe bournei* [43–45], suggesting that genes in the same subfamily may have different roles in abiotic stress responses and hormone-mediated signaling pathways. *EgrGRAS39* in the PAT1 subfamily were up-regulated more than 5-fold in response to ABA, GA3 and cold treatment, suggesting that this gene was an important gene for *E. grandis* in hormone response and cold stress. Under cold stress, the other member of the same family (PAT1) also shows the similar pattern as EgrGRAS39. The expression level of *EgrGRAS39* and *EgrGRAS34* were strongly up-regulated and peaked at 12 h. Members of the PAT1 subfamily not only play important roles in photosensitive pigment signalling, but also directly affect plant stress tolerance, for example, *VaPAT1* and *BdGRAS* genes, which have been reported in this subfamily, can positively respond to low temperature stress [43, 46]. With the exception of *EgrGRAS51*, all 18 *EgrGRAS* genes showed significant differences in expression after low-temperature stress treatment compared with normal conditions. The work showed that the expression level of *PbGRAS14* in *Phoebe bournei* increased significantly under low temperature stress [44]. Huang et al. also found that *SlGRAS1*, *SlGRAS3* and *SlGRAS4* in tomato can actively respond to low temperature stress, indicating that these genes were widely involved in low temperature resistance [23]. DELLA proteins are key negative regulators of GA_3_ signalling, and the expression patterns of the two DELLA subfamily genes in this study showed opposite trends after GA_3_ treatment. Among them, the expression level of *EgrGRAS51* was significantly up-regulated after drought treatment. In previous work, *BnaA6.RGA*, *BnaA9.RGA*, and *BnaC9.RGA* were induced by drought in Brassica napus, and BnaA6. RGA involved in the regulation of drought tolerance [47].

## Materials and methods

### Plant materials, growth conditions, and stress treatments

*E. grandis* GL1 clone plants were grown in pots using black soil and vermiculite. Under 14/10 h light/dark conditions, seedlings were grown in a greenhouse at 23–27 °C and 70% humidity. In the subsequent experiments, the plant material was cultivated for 3 months with watering every three days.

For the hormone stress treatments, *E. grandis* GL1 clone plants were irrigated with 300 mL of 100 µM ABA or 100 µM GA_3_ solution, and leaves were sampled at five time points (1, 6, 12, 24, and 168 h) after treatment.

For salinity and drought treatments, seedlings irrigated with 300 mL of 300 mM NaCl and 20% polyethylene glycerol-6000 (PEG) solution, respectively. All leaves were harvested at 0, 1, 6, 12, 24, and 168 h after each treatment.

For the low temperature treatment, seedlings were kept in a growth chamber at 4 °C and sampled at five time points (1, 6, 12, 24, and 168 h) after treatment. Untreated seedlings were used as controls. After each treatment, leaves were collected to be quickly frozen in liquid nitrogen and kept at -80 °C for total RNA extraction. Three biological and three technical replicates were employed.

### Sequence retrieval and gene identification

Protein sequences, CDS sequences, and annotation files for the plants *Arabidopsis*, rice, soybean, and *E. grandis* were downloaded from Phytozome databases. The Hidden Markov Model (HMM) file PFAM-A was downloaded from the Pfam database. The GRAS HMM (PF03514) was used to conduct an HMM-search in the genome of *E. grandis*, and the putative GRAS members were initially obtained. NCBI-CDD batch and the SMART program were used to identify the conserved domain of the screened protein sequences. The length, isoelectric point, molecular weight of the protein was all examined using the web program ExPASy. Exon number and chromosomal distribution of *EgrGRAS* genes were determined using the gff3 file from *E. grandis*. Additionally, the subcellular localization of EgrGRAS proteins was predicted by the WoLF PSORT online program (https://wolfpsort.hgc.jp/).

## Multiple alignment and phylogenetic analysis

Multiple alignments were carried out using ClustalX 2.11 with default parameters based on GRAS protein sequences from *Arabidopsis thaliana*, rice, soybean, and *E. grandis* [48]. After aligning the sequences, phylogenetic analysis was performed on them using the Neighbor Joining (NJ) method in MEGA 11 with 1000 bootstrap repetitions [49].

### Motif prediction and gene structure analysis

To study the conserved motifs of GRAS proteins of *E. grandis*, the identified GRAS proteins were uploaded to MEME (Multiple Em for Motif Elicitation) program to search their conserved motifs. The maximum number of motifs was set to 20 and the other parameters as default. The GFF3 annotation file was downloaded from Phytozome database, then the exon and intron location information of GRAS gene was extracted from the file. The online GSDS 2.0 (Gene Structure Display Server) website is used as a drawing.

### Collinearity and Ka/Ks analysis

From the Phytozome database, genome annotation files for *Arabidopsis*, rice, and soybean were retrieved. The genome-wide collinearity between willow and three other species was examined using MCScanX software, and the collinear results were mapped using TBtools software. TBtools was used to determine the ratio of non-synonymous to synonymous substitutions (Ka/Ks) of orthologues and paralogues [50].

### *Cis*-regulatory elements analysis

The GFF3 file and genome sequence were used to extract a 2 kb sequence upstream of the start codon of the *EgrGRAS* gene, which was then submitted to the PlantCARE website for *cis*-element analysis and identification.

### RNA extraction and quantitative real-time PCR (qRT-PCR)

Total RNA was extracted from each sample using the Aidlab Plant RNA Kit (Aidlab Biotech, Beijing, China). All RNAs were tested for concentration and integrity using electrophoresis and NanoDrop™ One/OneC (ThermoFisher SClentific, USA). The first-strand cDNA was synthesized using the Prime Script^TM^RT reagent Kit with gDNA Eraser (TaKaRa, Dalian, China). The *EF1α* gene was used as the reference gene [51]. Gene-specific primers were designed and checked for specificity using Primer Premier 5.0 and the TBtools, respectively. (Table S1). Real-time PCR was performed on a CFX96™ Real-Time System (BIO-RAD, California, USA) by using TB Green Premix Ex Taq II (Tli RNaseH Plus; TaKaRa Biotechnology) with a 10 µL sample volume. For each sample, we conducted three biological and three technical replicates. The relative expression levels of each gene were calculated as 2^−∆∆CT^ (∆C_T_ = C_T_, _target_ - C_T, CYP2_. ∆C_T_ = ∆C_T, treatment_ - ∆C_T, CK (0 h)_) compared with untreated control plants that were set as 1 [52]. the significance variance of treatments analysed and plotted using GraphPad software [53].

### Statistical and pearson correlation analysis

Statistical significance was performed using a paired Student’s *t* test. The mean values and standard deviations (SD) of three replicates were presented, and significant differences relative to controls were indicated at ^∗^*P* ≤ 0.05 and ^∗∗^*P* ≤ 0.01. Pearson correlation coefficients (PCCs) and p-values were obtained for qRT-PCR results using the R package and plotted. For the co-regulatory network, gene pairings with PCC values greater than 0.5 and significant at the 0.05 significance level (P-value) were collected. The co-regulatory networks were constructed in Cytoscape based on the PCCs of these gene pairs.

## Conclusion

In conclusion, a comprehensive analysis of the *GRAS* genes family in *E. grandis* was performed, including a genome-wide identification, characterization, and expression pattern. A total of 82 *EgrGRAS* genes had been identified, which can be divided into 9 subfamilies. Moreover, the duplication events have contributed to the expansion of the *GRAS* genes family in *E. grandis*. The results showed that the *EgrGRAS* gene family regulates multiple responses either positively or negatively. Particularly, the expression level of *EgrGRAS13* was strongly upregulated under both hormonal and stress treatments. This study laid a foundation for further research on the function of *GRAS* gene in *E. grandis* involved in hormone signal transduction and stress response.

### Electronic supplementary material

Below is the link to the electronic supplementary material.

Supplementary Material 1

Supplementary Material 2

## Data Availability

The genome sequences of *A. thaliana*, rice, soybean and *E. grandis* were downloaded from Phytozome database (https://phytozome-next.jgi.doe.gov/). The datasets supporting the results of this article are included in the article and Additional files.
