# Supplementary material for: Genome-wide identification and expression analysis of GRAS gene family in Eucalyptus grandis
Source: BMC Plant Biol. 2024 Jun 18;24:573. doi: 10.1186/s12870-024-05288-x (PMC11184746; doi:10.1186/s12870-024-05288-x)
Supplement: Supplementary file 2 — Supplementary Material 2 [file 12870_2024_5288_MOESM2_ESM.docx]

**Supplementary Figures**


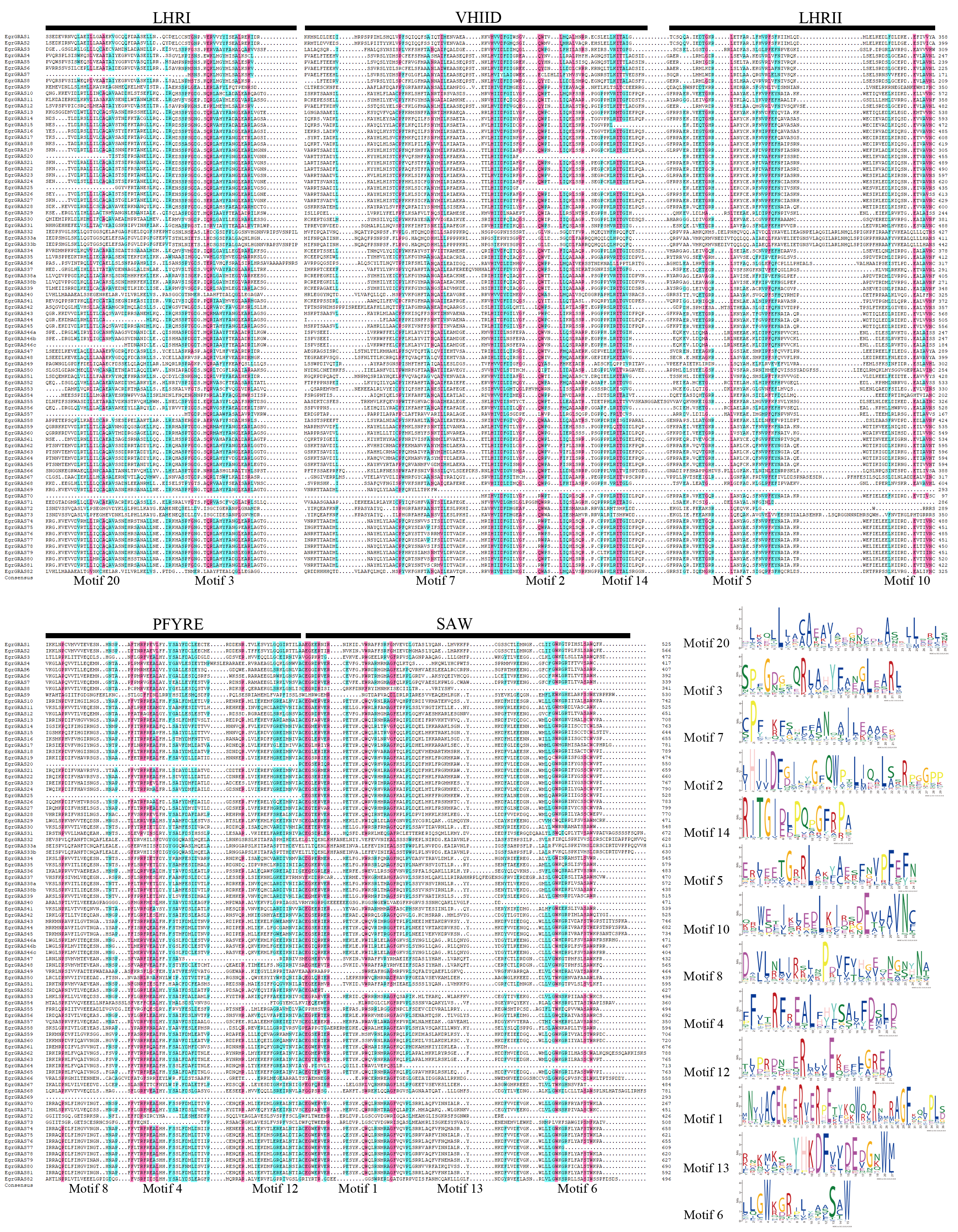


**Figure S1 Multiple sequence alignment of *GRAS* genes in *E. grandis*. Sequences were aligned using DNAMAN 8 software.**
